# Supplementary material for: The Role of MECP2 and CCR5 Polymorphisms on the Development and Course of Systemic Lupus Erythematosus
Source: Biomolecules. 2020 Mar 24;10(3):494. doi: 10.3390/biom10030494 (PMC7175371; doi:10.3390/biom10030494)

Values of AST concentrations in patients with HET +HOM∆32 genotype vary in small range in contrast with HOM genotype. Furthermore, AST mean values in CCR5 in HOM genotype in CCR5 gene have much more higher range of measurement mistake in comparison with HET+HOM∆32 genotype. The box plot for AST with whiskers is shown in Figure S1.

**Figure S1**. Box plot relative to groups – AST levels in patients with different CCR5 genotypes.


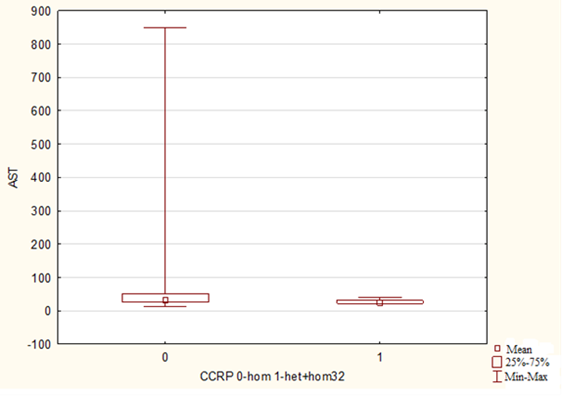

Supplement: Supplementary file 1 [file biomolecules-10-00494-s001.zip › Figure S1.docx]
