# Supplementary material for: The Role of MECP2 and CCR5 Polymorphisms on the Development and Course of Systemic Lupus Erythematosus
Source: Biomolecules. 2020 Mar 24;10(3):494. doi: 10.3390/biom10030494 (PMC7175371; doi:10.3390/biom10030494)
Supplement: Supplementary file 1 [file biomolecules-10-00494-s001.zip › Table S1.docx]

**Table S1**. The minor allele frequency (MAF) of four chosen MECP2 SNPs: rs2075596, rs173478, rs17435, rs223946 and for CCR5 polymorphism: rs333.

| **SNP ID** | **Allele** | **Type of SNP** | **MAF** | | |
| --- | --- | --- | --- | --- | --- |
|  |  |  | **SLE** | **Controls** | **1000Genomes – EU** |
| MECP2 rs2075596 | A>C / A>G | Intron Variant | 0.20 | 0.14 | 0.14 |
| MECP2 rs1734787 | A>C | Intron Variant | 0.19 | 0.18 | 0.15 |
| MECP2 rs17435 | T>A / T>C | Intron Variant | 0.29 | 0.22 | 0.19 |
| MECP2 rs2239464 | G>A | Intron Variant | 0.26 | 0.22 | 0.19 |
| CCR5 rs333 | 32 bp deletion | Intron Variant | 0.12 | 0.13 | 0.11 |
