# Supplementary material for: The Role of MECP2 and CCR5 Polymorphisms on the Development and Course of Systemic Lupus Erythematosus
Source: Biomolecules. 2020 Mar 24;10(3):494. doi: 10.3390/biom10030494 (PMC7175371; doi:10.3390/biom10030494)
Supplement: Supplementary file 1 [file biomolecules-10-00494-s001.zip › Table S1.pdf]

Table S1. The minor allele frequency (MAF) of four chosen MECP2 SNPs: rs2075596, rs173478, rs17435, rs223946 and for CCR5 polymorphism: rs333.

SNP ID	Allele	Type of SNP	MAF		
			SLE	Controls	1000Genomes – EU
MECP2 rs2075596	A>C / A>G	Intron Variant	0.20	0.14	0.14
MECP2 rs1734787	A>C	Intron Variant	0.19	0.18	0.15
MECP2 rs17435	T>A / T>C	Intron Variant	0.29	0.22	0.19
MECP2 rs2239464	G>A	Intron Variant	0.26	0.22	0.19
CCR5 rs333	32 bp deletion	Intron Variant	0.12	0.13	0.11
