# Supplementary figures and images for: The Role of MECP2 and CCR5 Polymorphisms on the Development and Course of Systemic Lupus Erythematosus
Source: Biomolecules. 2020 Mar 24;10(3):494. doi: 10.3390/biom10030494 (PMC7175371; doi:10.3390/biom10030494)

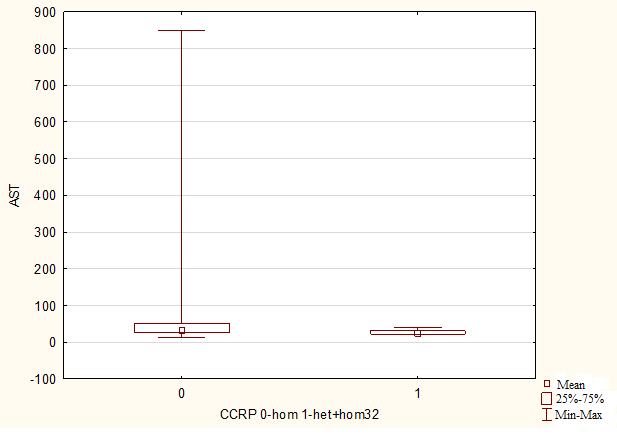

Supplement: Supplementary file 1 [file biomolecules-10-00494-s001.zip › Figure S1.png]
